# Voice analyses using smartphone-based data in patients with bipolar disorder, unaffected relatives and healthy control individuals, and during different affective states

**DOI:** 10.1186/s40345-021-00243-3

**Published:** 2021-12-01

**Authors:** Maria Faurholt-Jepsen, Darius Adam Rohani, Jonas Busk, Maj Vinberg, Jakob Eyvind Bardram, Lars Vedel Kessing

**Affiliations:** 1 Copenhagen Affective Disorder Research Center (CADIC), Psychiatric Center Copenhagen, Rigshospitalet, Blegdamsvej 9, 2100 Copenhagen, Denmark; 2 Department of Health Technology, Technical University of Denmark, Lyngby, Denmark; 3 Department of Energy Conversion and Storage, Technical University of Denmark, Lyngby, Denmark; 4 Psychiatric Centre North Zealand, Hilleroed, Denmark

**Keywords:** Voice analysis, Classification, Random Forest, Bipolar disorder, openSMILE

## Abstract

**Background:**

Voice features have been suggested as objective markers of bipolar disorder (BD).

**Aims:**

To investigate whether voice features from naturalistic phone calls could discriminate between (1) BD, unaffected first-degree relatives (UR) and healthy control individuals (HC); (2) affective states within BD.

**Methods:**

Voice features were collected daily during naturalistic phone calls for up to 972 days. A total of 121 patients with BD, 21 UR and 38 HC were included. A total of 107.033 voice data entries were collected [BD (n  = 78.733), UR (n  = 8004), and HC (n  =  20.296)]. Daily, patients evaluated symptoms using a smartphone-based system. Affective states were defined according to these evaluations. Data were analyzed using random forest machine learning algorithms.

**Results:**

Compared to HC, BD was classified with a sensitivity of 0.79 (SD 0.11)/AUC  = 0.76 (SD 0.11) and UR with a sensitivity of 0.53 (SD 0.21)/AUC of 0.72 (SD 0.12). Within BD, compared to euthymia, mania was classified with a specificity of 0.75 (SD 0.16)/AUC  =  0.66 (SD 0.11). Compared to euthymia, depression was classified with a specificity of 0.70 (SD 0.16)/AUC  =  0.66 (SD 0.12). In all models the user dependent models outperformed the user independent models. Models combining increased mood, increased activity and insomnia compared to periods without performed best with a specificity of 0.78 (SD 0.16)/AUC  =  0.67 (SD 0.11).

**Conclusions:**

Voice features from naturalistic phone calls may represent a supplementary objective marker discriminating BD from HC and a state marker within BD.

## Introduction

Bipolar disorder (BD) is characterized by recurrent affective episodes with significant alterations in core features of mood, activity and sleep (Goodwin and Jamison 1996). There is a substantial diagnostic delay and a progression of illness severity during untreated years, stressing the need for earlier diagnosis and intervention (Baldessarini et al. 2007; Kessing et al. 2014). However, due to the lack of objective tests, the diagnostic process as well as the clinical assessment of illness activity relies on patient information, clinical evaluation and rating scales (Phillips and Kupfer 2013). This evaluation process involves a risk of individual observer bias and recall distortions at various levels (Silva et al. 2015; Silva et al. 2016). Therefore, objective supplementary methods for assisting clinicians in the diagnosis and the assessment of illness activity in BD would be a tremendous advantage.

It is well established that 20–30% of unaffected first-degree relatives (UR) of patients with BD develop affective illness, compared to 2–5% among healthy control individuals (HC) (Vedel Kessing et al. 2021). Further, functioning is substantially and broadly decreased within education, employment, income, cohabitating and being married in UR compared with HC (Sletved et al. 2021). Therefore, it is likely that UR to patients with BD will show alterations in prodromal symptoms and features related to illness intermediate between patients with BD and HC.

Speech is individual for each person like ‘a fingerprint’, and speech patterns have shown to provide indicators of mental disorders. In 1921, Emil Kraepelin emphasized that patients with depression tended to have lower pitch, lower speech rate and more monotonous speech (Lord et al. 1921), and studies analyzing the spoken language in affective disorders date back as early as 1938 (Newman and Mather 1938). Differences in language structure between patients with BD and HC have been described, and changes in speech has been suggested as objective, sensitive and valid measures of depressive and (hypo-)manic episodes in BD (Mundt et al. 2012; Raucher-Chéné et al. 2017; Arevian 2020). A recent systematic review concerning automated assessment of psychiatric disorders using speech suggested that speech processing technology could aid mental health assessments (Low et al. 2020). However, this review also addressed obstacles concerning the lack of larger, transdiagnostic and longitudinal studies (Low et al. 2020).

Digital phenotyping refers to approaches in which personal data gathered from mobile devices and sensors is analyzed to provide health information on physiological functions, or behavioral indicators, such as the user’s speech (Insel 2017; Ebner-Priemer 2020). These data can be seen as digital footprints/digital markers—or data traces arising as a by-product from interactions with technology. Software for ecologically extracting data on voice features from naturalistic phone calls has been developed (Eyben et al. 2010). Previous studies concerning voice features collected digitally within BD have investigated the use of speech to classify affective states and suggested that (hypo)manic states more accurately were classified than depressive states (Karam et al. 2014; Muaremi et al. 2014; Maxhuni et al. 2016; Gideon et al. 2016; Zhang et al. 2018; Vanello et al. 2012; Guidi et al. 2015). A previous study conducted by the authors found, that voice features collected in naturalistic settings using smartphones may represent objective state markers in patients with BD (Faurholt-Jepsen 2016). However, this previous study included a small number of patients and thus hold less strength. Moreover, it has not been investigated whether voice features collected from naturalistic phone calls can discriminate between BD, UR and HC. Smartphone-based voice technology could potentially aid clinicians in early diagnosing of BD and in identifying and targeting prodromal symptoms and states in UR.

## Objectives

The present study aimed to investigate whether voice features collected from naturalistic phone calls (1) could discriminate between patients with BD, UR, and HC; (2) within patients with BD could discriminate between (a) mania and euthymia and (b) depression and euthymia; and (3) within patients with BD could discriminate between (a) periods with increased activity and neutral activity, (b) periods with decreased activity and neutral activity, (c) periods with insomnia and periods without, and (d) periods with combined increased mood, increased activity and insomnia and periods without. We hypothesized that voice features would be able to discriminate between patients with BD and HC, and between UR and HC (with UR intermediate between patients with BD and HC), and further discriminate between states within patients with BD.

## Methods and materials

### Study design and participants

The present study included data from two studies—the RADMIS trial (Faurholt-Jepsen et al. 2020) and the larger ongoing Bipolar Illness Onset study (BIO study) (Kessing 2017). Data were collected during the period from 2017 to 2020. All participants underwent The Schedules of Clinical Assessment in Neuropsychiatry (SCAN) interview (Wing et al. 1990) to confirm the clinical diagnosis of (or the lack of) BD.

#### The RADMIS trial

Patients with a diagnosis of BD who were hospitalized due to an affective episode and being discharged from one of five psychiatric centers at the Mental Health Services, Capital Region of Denmark, Denmark in the period from May 2017 to August 2019 were invited to participate in the RADMIS trial. Inclusion criteria: age above 18 years, BD diagnosis (ICD-10), discharge from a psychiatric hospital in The Capital Region of Denmark following an affective episode (depression, mania or mixed episode). Exclusion criteria: pregnancy and a lack of Danish language skills. In addition to standard treatment, patients were randomized with a balanced allocation ratio to either (1) daily use of a smartphone-based monitoring system (the Monsenso system—se description below) (the intervention group) or to (2) normal use of smartphones (the control group) during a 6 months follow-up period. Only patients from the intervention group providing smartphone-based data were included in the present study.

#### The BIO study

Three groups of participants were included in the BIO study: patients with newly diagnosed BD, UR, and HC.

#### Patients with BD

Inclusion criteria: a newly diagnosis of a single manic episode or BD (ICD-10) and ages between 15 and 70 years.

#### UR

UR, siblings or children, to the patients included in the BIO study, were recruited after permission from patients. Exclusion criteria: any previous or current psychiatric diagnosis lower than F34.0 (CD-10) (i.e., organic mental disorders, mental and behavioral disorders due to psychoactive substance use including alcohol, schizophrenia or other psychotic disorders, affective disorders).

#### HC

HC were recruited among blood donors, aged between 15 and 70 years, from the Blood Bank at Rigshospitalet, Copenhagen. Exclusion criteria: treatment requiring psychiatric disorder in the individual or one of the individuals’ first-degree family members. All participants in the BIO study were offered to use a smartphone-based monitoring system on a daily basis (the Monsenso system—see description below) during the study period.

### Clinical assessments

Clinical evaluations of the severity of depressive and manic symptoms were conducted by a trained researcher using the Hamilton Depression Rating Scale 17-items (HDRS) (Hamilton 1967) and the Young Mania Rating Scale (YMRS) (Young et al. 1978).

### Patient-reported smartphone-based data

A smartphone-based monitoring system (the Monsenso system) was installed on the participants own smartphones (both iPhone and Android smartphones). The smartphone-based monitoring system developed by the authors was used by the patients with BD on a daily basis to collect fine-grained real-time recordings of mood, activity, and sleep duration (Bardram et al. 2013). Mood was evaluated with scores on a 9-point scale ranging from depressed to manic (− 3, − 2, − 1, − 0.5, 0, 0.5, 1, 2, 3). Euthymia mood was defined a priori as a mood score of − 0.5, 0, 0.5. Depression was defined as mood score  < − 0.5, and mania was defined as mood score  >  0.5. Daily activity levels were rated on a 7-point scale (− 3, − 2, − 1, 0, 1, 2, 3) with 0 representing normal activity level. Sleep duration was calculated based on daily reports of bedtime and wake-up time. Insomnia was defined as total sleep duration  <  360 min. In addition, a broader definition of mania was made by combining increased mood (> 0.5), increased activity (> 0) and decreased sleep (< 360 min.).

### Voice features

Voice features were collected from the participants’ phone calls (only Android smartphones) during their everyday life using the open-source Speech and Music Interpretation by Large-space Extraction (openSMILE vs. 2.1.0, Emo-Large) toolkit (Eyben et al. 2010; Schuller et al. 2010). The toolkit is a feature extractor for signal processing and machine learning applications, and it is designed for real-time processing. The toolkit used a built-in voice activity detection to live record voice samples from each incoming and outgoing phone call on the participants’ smartphone. The voice activity detection was run solely on the study participants’ onboard microphone such that the voice segments represented one recorded audio stream from the participant's voice. The audio stream was used to extract acoustic features ‘online’, e.g., directly on the study participants’ smartphones for each phone call. Voice samples were deleted locally on the smartphone after each phone call, and thus there was not access to any content related material from phone calls. The Emo-Large was a predefined set consisting of 6552 features, e.g., pitch, loudness, and energy, represented through various 1st level descriptive statistics including means, regression coefficients, and percentiles. The set has been found to be particularly relevant for classifying emotions (Pfister and Robinson 2010).

### Statistical methods

Data were imported to and processed in Python (version 3.8) with packages sklearn (v. 0.23.2), imblearn (v. 0.7.0), and pandas (v. 1.1.4).

Aim 1 concerned the discrimination between patients with BD, UR, and HC based on the use of collected voice features. Aims 2 and 3 concerned the use of voice data from patients with BD to classify the symptom class labels within mood, energy, and sleep collected daily from smartphones, and a combination of the three.

For all analyses Random Forest (RF) classifiers were built to discriminate between classes (Breiman 2001). The RF classifiers combine several decision tree classifiers into a single classifier. A RF model uses the ensemble technique to yield a prediction from multiple independent decision tree classifiers. RF models were chosen as they generally can handle large number of features while being robust to overfitting. Each tree is generated from a subsample of the data and using a random subset of features to ensure maximal degree of independence among the trees. The classifier uses supervised learning, i.e., information of the group status/affective state, to build nodes that split the dataset into groups. These splits continue until the model either has a group with only a single class, or if further splits are unable to improve the classification. Call entries with missing voice feature values and features with identical values (i.e., zero variance) were removed.

All classifications were binary (e.g., patients with BD versus HC). For aim 2 and 3 patient-reported smartphone-based data for any specific day during the study period were included in the analyses if both voice features and patient-reported smartphone-based on mood, activity or sleep were available for the same day. We evaluated RF models on the resulting data set through a five-fold participant-based cross-validation. Five-fold cross-validation partitions the data in 5 parts of approximately the same size. Five to one partitions of the data were used to train the model, while the last partition was used to test the model, thereby evaluating the performance on unseen data samples. This was repeated 5 times so all samples were used for testing once to yield an average performance across all folds. We used a participant-based cross-validation version, where the test partition included participants that were not part of the training partition and vice versa. The participant-based method is particularly important for aim 1 since all voice data for each participant is identically labeled (i.e., either BD, UR, or HC). If the same participant is represented in the training and test partition the model would falsely learn to discriminate participant-based characteristics instead of clinical diagnose or state. Ad-hoc analyses without the participant-based cross-validation displayed significant better results. Therefore, to avoid learning on participant traits, all analyses included participant-based cross-validation.

In each cross-validation fold, the training set was used to calculate standardization parameters that transform the voice features training set to zero mean, unit variance. The calculated parameters were then applied to the test set. We used this standardization approach to create an unbiased data transformation invariant for factors such as gender, age, or microphone types selected by the phone vendors. As we used a participant-based cross-validation approach, the standardization was done for each voice feature across all participants.

Analyses concerning aims 2 and 3 were separated in two model types. First, a user-independent model that—as for aim 1—combines data from all participants in the same model. The model uses information from known participants to classify symptoms of unknown patients. Second, a user-dependent model personalized model for each patient was built.

We observed significant class imbalance in the data for all aims (e.g., fewer cases of symptoms of ‘mania’ compared with ‘euthymia’). Therefore, we applied a resampling process on the training data to balance the two classes. We did a combination with SMOTE oversampling (Chawla et al. 2002) of the minority class to represent 33% of the cases, followed by random under sampling of the majority class until the sample size was identical to the minority class. The combination of oversampling with SMOTE and under sampling has previously been shown effective to counter class imbalance (García et al. 2016). Without a resampling scheme, the RF classifier would favor overrepresented classes. However, resampling was only performed on the training data, to keep the test set class distribution representative for the collected data. In the cases where class distribution was less than 33% skewed, we only performed random under sampling.

### Classifier performance

We applied several standard metrics for binary classification computed on a test set held out data and compared the results to a majority vote baseline model.

The metrics included a) ‘accuracy’ (defined as the number of correct classifications of the positive and negative cases divided by the total number of cases); (b) ‘F1-score’ (estimates the model’s ability to identify the positive class correctly, and was defined as the true positives divided by the true positives and the average between false positives and false negatives); (c) ‘sensitivity’ (defined as true positives divided by positives); (d) ‘specificity’ (defined as true negatives over all negatives); (e) ‘area under the characteristic curve’ (AUC) which is the area under the entire Receiver Operating Characteristic (ROC) curve. A ROC curve displays the model performance of sensitivity and specificity at all probability thresholds. The sensitivity and specificity reported in the tables are based on a threshold of 50%. An AUC value of 0.5 represents random guessing, while a value of one is a perfect classifier. To further strengthen performance interpretation a Bayesian inference framework with intrinsic priors was added (B10) (Leon-Novelo et al. 2012). The method handles unbalanced data well as proven through various simulated and real work examples (Olivetti et al. 2015). The measure is based on a statistical foundation through a test of statistical independence between, here, our predicted results and the actual symptom registered. Therefore, a direct standardized guideline exists. A value below 0 indicates a negative evidence for a statistically dependency, a value between 1 and 3 suggests a more positive indication, 3–5 a strong indication, while a value above 5 is a decisive indication of statistically dependence.

All classification metrics were computed within each cross-validation fold to yield a mean (M) and standard deviation (SD) value across all five-folds. In the personalized model we further averaged across all patients.

**Fig. 1:**
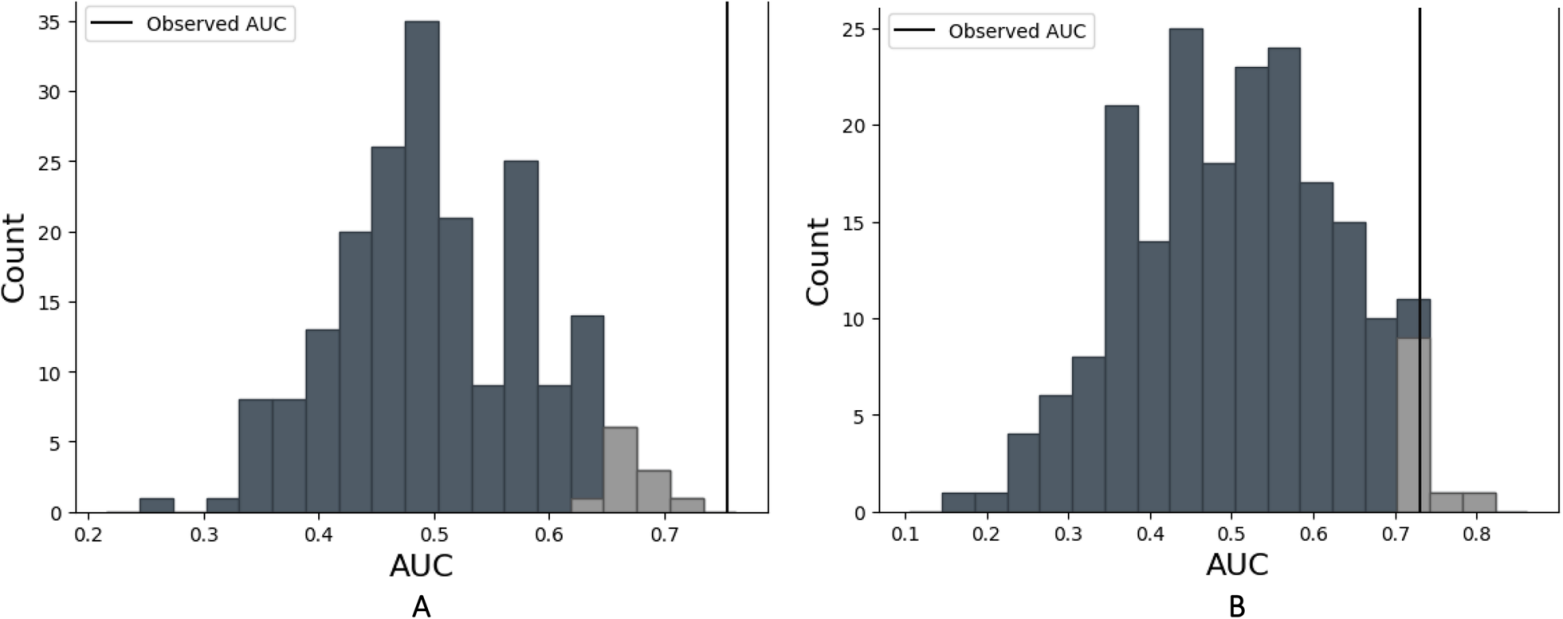
A generated null distribution of AUC values from a permutation test where the class labels (e.g., patients with bipolar disorder and healthy controls) are randomly shuffled 200 times and an AUC value for each permutation is plotted. The light grey region represents the critical area with the 5% largest values. The vertical lines represent the observed AUC values from the true class labels. **A** Generated null-distribution for the Random Forest classification of patients with bipolar disorder against healthy control individuals. **B** Generated null-distribution for the Random Forest classification of patients with bipolar disorder against unaffected relatives

For aim 1, we ran a randomized permutation model (Berry et al. 2002) to test whether voice data from the three populations were statistically significantly different from each other. We randomly shuffled the class label for each participant and re-ran the entire RF classification. This was repeated 200 times to generate a non-parametric null-distribution of AUC scores (Fig. 1). Statistically significance was determined if the RF test AUC statistics with true class labels exceeds the null distribution with a significance level of p  = 0.05.

For aims 2 and 3, we developed a majority vote model and a random classifier as a baseline. Unlike the RF model, the baseline models did not include voice data. Simply, in the majority vote model, the most frequently observed class label in the training data, was used to classify test data. In cases where there was an equal class distribution, the test data was classified at random. The random classifier used a uniform distribution to randomly choose a class label.

### Ethical considerations

#### The RADMIS trial

The RADMIS trial was approved by the Regional Ethics Committee in The Capital Region of Denmark and the data agency, Capital Region of Copenhagen (H-16046093, RHP-2017-005, I-Suite: 05365) and registered at ClinicalTrials.gov (NCT03033420).

#### The BIO study

The study protocol was approved by the Committee on Health Research Ethics of the Capital region of Denmark (protocol No. H-7-2014-007) and the Danish Data Protection Agency, Capital Region of Copenhagen (RHP-2015-023).

Both studies complied with the Declaration of Helsinki (Seoul, October 2008). All participants provided written informed consent. Data from smartphones were stored by Monsenso subject to a data management agreement between Monsenso and The Capital Region of Denmark.

## Results

### Background characteristics

Overall, a total of 254 participants were included in the present study. A total of 27 participants dropped out of the study (mainly due to not having the time to participate), and a total of 47 participants did not provide voice data. Thus, a total 180 participants were available and included in the present report (patients with BD n  = 121, UR n  = 21, and HC n  = 38). The participants provided on average 157 (SD  = 174) days with at least one voice recording with a range between 1 and 972 days.

A total of 107.033 voice data entries were collected across patients with BD (n  = 78.733), UR (n  = 8004), and HC (n  = 20.296).

Overall, the participants had a mean age of 34.5 (SD 11.5) years with a range from 18 to 67 years. A total of 56% (n  = 101) were women.

A total of 41% (n  = 49) of patients with BD had a HDRS score  ≥  13 at inclusion, and a mean score on the YMRS of 3.76 (SD 4.71). Only 5% (n  = 6) of the patients had an YMRS score  ≥ 13 at inclusion. There were no statistically significant differences in age or sex distribution across the three populations. There was a statistically significant difference in education level (BD: HC, p  = 0.03) and unemployment (BD: HC, p  = 0.001; BD: UR, p  = 0.021) between patients with BD, HC and UR. Further background characteristics are presented in Table 1.

**Table 1:** Background characteristics of participants, n  = 180

	Patients with bipolar disorder (BD)	Healthy control individuals (HC)	Unaffected relatives (UR)	p
n, % female	121 (60.0)	38 (45.0)	21 (52.0)	p > 0.16
Age	35.71 (12.35)	31.66 (10.87)	32.29 (10.57)	p > 0.16
Employed, % (n)	17 (20)	50 (19)	57 (12)	BD:HC (p = 0.002)
				UR:HC (p = 0.88)
Student, % (n)	31 (37)	39 (15)	33 (7)	p > 0.42
Unemployed, % (n)	30 (36)	8 (3)	10 (2)	BD:HC (p = 0.001)
				UR:HC (p = 0.76)
Education (years)	13.68 (4.66)	15.58 (1.57)	15.07 (2.62)	BD:HC (p = 0.03)
				UR:HC (p = 0.89)
Bipolar subtype I, % (n)	38 (46)	N/A	N/A	N/A
HAMD at inclusion	10.84 (6.93)	0.95 (1.62)	3.10 (3.32)	BD:HC (p < 0.001)
				UR:HC (p = 0.38)
YMRS at inclusion	3.76 (4.71)	0.51 (0.98)	1.25 (1.92)	BD:HC (p < 0.001)
				UR:HC (p = 0.77)
Previous hospitalizations (number)	4 [1–50]	N/A	N/A	N/A
Previous depressive episodes (number)	10 [1–80]	N/A	N/A	N/A
Previous (hypo)manic episodes (number)	14 [1–182]	N/A	N/A	N/A
Illness duration (years)	14.86 (10.37)	N/A	N/A	N/A
Psychotropic medication				
Anticonvulsant, % (n)	42 (51)	N/A	N/A	N/A
Lithium, % (n)	48 (58)	N/A	N/A	N/A
Antipsychotics, % (n)	49 (59)	N/A	N/A	N/A
Antidepressants, % (n)	18 (22)	N/A	N/A	N/A

Data are mean (SD), median [IQR] or proportions (%, n) unless otherwise stated

*HAMD* Hamilton Depression Rating Scale 17-items score

*YMRS* Young Mania Rating Scale score

### Classification of groups

Table 2 present the results for classification of patients with BD (78.731 observations), HC (20.296 observations) and UR (8004 observations) based on voice features.

**Table 2:** Discrimination between patients with bipolar disorder (BD) (n  = 121), unaffected relatives (UR) (n  = 21) and healthy control individuals (HC) (n  = 38) based on voice features collected from smartphones, n  = 180

Binary classifier (n = number of observations)	Model type	Accuracy (SD)	F1 score (SD)	Sensitivity (SD)	Specificity (SD)	AUC (SD)
BD (n = 78,731) compared with HC (n = 20,296)	Random Forest model	0.72 (0.09)	0.81 (0.07)	0.79 (0.11)	0.54 (0.20)	0.76 (0.11)
	Majority vote	0.67 (0.00)	0.88 (0.06)	1.0 (0.00)	0.00 (0.00)	0.50 (0.00)
BD (n = 78,731) compared with UR (n = 8004)	Random Forest model	0.68 (0.049)	0.81 (0.03)	0.73 (0.07)	0.28 (0.11)	0.52 (0.09)
	Majority vote	0.95 (0.00)	0.95 (0.02)	1.00 (0.00)	0.00 (0.00)	0.50 (0.00)
UR (n = 8004) compared with HC (n = 20,296)	Random Forest model	0.59 (0.13)	0.38 (0.15)	0.53 (0.21)	0.67 (0.24)	0.72 (0.12)
	Majority vote	0.80 (0.00)	0.00 (0.00)	0.00 (0.00)	1.00 (0.00)	0.50 (0.00)

The sensitivity and specificity for classifying patients with BD versus HC was 0.79 (SD 0.11) and 0.54 (SD 0.20), respectively and with an AUC of 0.76 (SD 0.11). The sensitivity and specificity for classifying patients with BD versus UR was 0.73 (SD 0.07) and 0.28 (SD 0.11), respectively with an AUC of 0.52 (SD 0.09). The sensitivity and specificity for classifying UR versus HC was 0.53 (SD 0.21) and 0.67 (SD 0.24), respectively and with an AUC of 0.72 (SD 0.12). Figure 1A, B presents the generated null-distribution of AUC scores from permuted class labels as generated from the randomized permutation model. The lighter area shows the critical level for a one-tail test with a significance level of 0.05, e.g., values that are high enough to be considered statistically significant at the 0.05 level. The horizontal line represent the observed AUC value from Table 2 for the correct class labels. In both cases, the observed AUC for patients with BD versus HC (0/200, p  < 0.001) and UR versus HC (6/200, p  = 0.03) differed statistically significantly. Thus, there was a statistically significant difference in voice feature between patients with BD versus HC and a statistically significant difference in voice features between UR and HC.

### Classifications of states within bipolar disorder

A total of 100 patients with BD provided both voice features and smartphone-based patient-reported data. Table 3 present the results for classification of different states in patients with BD. In all the models presented in Table 3, the personalized user-dependent models outperformed the general user-independent models. Therefore, the results from the user-dependent models are presented below.

**Table 3:** Classification within patients with bipolar disorder (n  = 100) according to patient-reported smartphone-based data on mood, activity and sleep^a^

Binary classifier n = number of observations	Model type	Accuracy (SD)	F1 score (SD)	Sensitivty (SD)	Specificity (SD)	AUC (SD)	B10 (SD)^b^
Mood							
Mania (n = 1205) versus euthymia (n = 38,329)	Random Forest model-user independent	0.72 (0.18)	0.05 (0.03)	0.23 (0.12)	0.73 (0.19)	0.51 (0.07)	− 2.65 (0.34)
	Random Forest model-user dependent	0.74 (0.16)	0.25 (0.22)	0.42 (0.22)	0.75 (0.16)	0.66 (0.11)	0.53 (3.69)
	Majority vote-user independent	0.94 (0.00)	0.00 (0.00)	0.00 (0.00)	1.00 (0.00)	0.50 (0.00)	− 3.18 (1.61)
	Majority vote-user dependent	0.97 (0.00)	0.08 (0.25)	0.11 (0.31)	0.89 (0.31)	0.48 (0.02)	− 3.23 (1.35)
	Random model-user independent	0.49 (0.00)	0.06 (0.03)	0.49 (0.05)	0.50 (0.01)	0.50 (0.00)	− 2.82 (0.27)
	Random model-user dependent	0.49 (0.00)	0.15 (0.09)	0.52 (0.03)	0.48 (0.03)	0.50 (0.00)	− 2.21 (0.59)
Depression (n = 5329) versus euthymia (n = 38,329)	Random Forest model-user independent	0.63 (0.06)	0.21 (0.07)	0.40 (0.07)	0.66 (0.05)	0.55 (0.05)	− 1.82 (0.22)
	Random Forest model-user dependent	0.70 (0.13)	0.40 (0.21)	0.53 (0.22)	0.70 (0.16)	0.66 (0.12)	2.78 (5.49)
	Majority vote-user independent	0.89 (0.00)	0.00 (0.00)	0.00 (0.00)	1.00 (0.00)	0.50 (0.00)	− 3.78 (1.31)
	Majority vote-user dependent	0.71 (0.00)	0.11 (0.28)	0.13 (0.33)	0.88 (0.33)	0.49 (0.02)	− 3.29 (0.00)
	Random model-user independent	0.50 (0.00)	0.24 (0.06)	0.51 (0.01)	0.49 (0.00)	0.50 (0.00)	− 2.07 (0.16)
	Random model-user dependent	0.46 (0.0)	0.31 (0.17)	0.55 (0.07)	0..44 (0.05)	0.50 (0.00)	− 1.80 (0.46)
Activity							
Increased (n = 12,890) versus neutral (n = 21,661)	Random Forest model-user independent	0.46 (0.08)	0.43 (0.10)	0.55 (0.07)	0.41 (0.09)	0.48 (0.07)	− 1.38 (0.20)
	Random Forest model-user dependent	0.67 (0.13)	0.55 (0.25)	0.55 (0.26)	0.58 (0.24)	0.61 (0.10)	1.85 (5.92)
	Majority vote-user independent	0.48 (0.00)	0.00 (0.00)	0.00 (0.00)	1.00 (0.00)	0.50 (0.00)	− 3.52 (0.00)
	Majority vote-user dependent	0.57 (0.00)	0.45 (0.40)	0.56 (0.49)	0.44 (0.49)	0.49 (0.01)	− 0.02 (0.15)
	Random model-user independent	0.50 (0.00)	0.45 (0.07)	0.50 (0.01)	0.49 (0.01)	0.50 (0.00)	− 1.76 (0.07)
	Random model-user dependent	0.53 (0.00)	0.50 (0.13)	0.56 (0.05)	0.45 (0.05)	0.50 (0.00)	− 1.44 (0.63)
Decreased (n = 10,288) versus neutral (n = 21,661)	Random Forest model-user independent	0.50 (0.03)	0.42 (0.06)	0.58 (0.04)	0.47 (0.05)	0.54 (0.02)	− 1.43 (0.42)
	Random Forest model-user dependent	0.66 (0.11)	0.53 (0.18)	0.53 (0.20)	0.65 (0.21)	0.62 (0.10)	3.13 (6.40)
	Majority vote-user independent	0.69 (0.00)	0.00 (0.00)	0.00 (0.00)	1.00 (0.00)	0.50 (0.00)	− 3.04 (0.00)
	Majority vote-user dependent	0.71 (0.00)	0.27 (0.37)	0.35 (0.47)	0.65 (0.47)	0.49 (0.02)	− 0.02 (0.15)
	Random model-user independent	0.50 (0.00)	0.41 (0.07)	0.51 (0.01)	0.49 (0.01)	0.50 (0.00)	− 1.77 (0.10)
	Random modeluser dependent	0.55 (0.00)	0.45 (0.12)	0.55 (0.05)	0.44 (0.05)	0.50 (0.00)	− 1.39 (0.66)
Sleep							
Insomnia (n = 8474) versus normal sleep (n = 36,140)	Random Forest model-user independent	0.70 (0.05)	0.13 (0.03)	0.16 (0.10)	0.82 (0.09)	0.48 (0.04)	− 1.49 (0.43)
	Random Forest model-user dependent	0.70 (0.16)	0.33 (0.18)	0.39 (0.22)	0.73 (0.17)	0.59 (0.08)	0.28 (2.66)
	Majority vote-user independent	0.85 (0.00)	0.00 (0.00)	0.00 (0.00)	1.00 (0.00)	0.50 (0.00)	− 3.13 (1.17)
	Majority vote-user dependent	0.90 (0.00)	0.05 (0.17)	0.06 (0.24)	0.93 (0.24)	0.49 (0.01)	− 3.36 (1.19)
	Random model-user independent	0.50 (0.00)	0.25 (0.07)	0.50 (0.01)	0.49 (0.00)	0.50 (0.00)	− 2.03 (0.13)
	Random model-user dependent	0.48 (0.00)	0.32 (0.13)	0.55 (0.05)	0.44 (0.04)	0.50 (0.00)	− 1.77 (0.48)
A broader definition of mania							
Combined increased mood, activity and decreased sleep							
Combined increased mood, activity and decreased sleep (n = 471) versus rest (n = 43,243)	Random Forest model-user independent	0.77 (0.10)	0.03 (0.02)	0.29 (0.17)	0.77 (0.11)	0.58 (0.06)	− 1.68 (0.28)
	Random Forest model-user dependent	0.77 (0.15)	0.17 (0.16)	0.41 (0.21)	0.78 (0.16)	0.67 (0.11)	2.15 (6.02)
	Majority vote-user independent	0.98 (0.00)	0.00 (0.00)	0.00 (0.00)	1.00 (0.00)	0.50 (0.00)	− 3.44 (0.00)
	Majority vote-user dependent	0.99 (0.00)	0.00 (0.00)	0.00 (0.00)	1.00 (0.00)	0.48 (0.02)	− 3.43 (1.30)
	Random model-user independent	0.49 (0.00)	0.13 (0.05)	0.50 (0.00)	0.49 (0.00)	0.50 (0.00)	− 2.39 (0.18)
	Random model-user dependent	0.46 (0.00)	0.46 (0.18)	0.56 (0.02)	0.44 (0.04)	0.50 (0.00)	− 1.78 (0.24)

The number of observations is the recorded samples before any resampling or cross-validation partitioning

^a^Euthymia was defined as a mood score of − 0.5, 0, 0.5. Depression was defined as a mood score  <  − 0.5, and mania was defined as a mood score  > 0.5. Increased activity was defined as a score  > 0, and decreased activity was defined as a score  < 0. Insomnia was defined as total sleep duration  < 360 min. A broader definition of mania was made by combining increased mood, increased activity and decreased sleep

^b^Bayesian inference framework

#### Mania (1205 observations) versus euthymia (38.329 observations)

The sensitivity and specificity for classifying mania versus euthymia was 0.42 (SD 0.22) and 0.75 (SD 0.16), respectively and with an AUC of 0.66 (SD 0.11).

#### Depression (5329 observations) versus euthymia

The sensitivity and specificity for classifying depression versus euthymia was 0.53 (SD 0.22) and 0.70 (SD 0.16), respectively and with an AUC of 0.66 (SD 0.12).

#### Increased activity (12.890 observations) versus neutral activity (21.661 observations)

The sensitivity and specificity for classifying increased activity versus neutral activity was 0.55 (SD 0.26) and 0.58 (SD 0.24), respectively and with an AUC of 0.61 (SD 0.10).

#### Decreased activity (10.228 observations) versus neutral activity

The sensitivity and specificity for classifying decreased activity versus neutral activity was 0.53 (SD 0.20) and 0.65 (SD 0.21), respectively and with an AUC of 0.62 (SD 0.10).

#### Insomnia (8474 observations) versus periods without (36.140)

The sensitivity and specificity for classifying insomnia versus periods without was 0.39 (SD 0.22) and 0.73 (SD 0.17), respectively and with an AUC of 0.59 (SD 0.08).

#### Combined increased mood, increased activity and insomnia (471 observations) versus periods without (43.243 observations)

The sensitivity and specificity for classifying combined increased mood, increased activity and insomnia versus periods without was 0.41 (SD 0.21) and 0.78 (SD 0.16), respectively and with an AUC of 0.67 (SD 0.11).

**Fig. 2:**
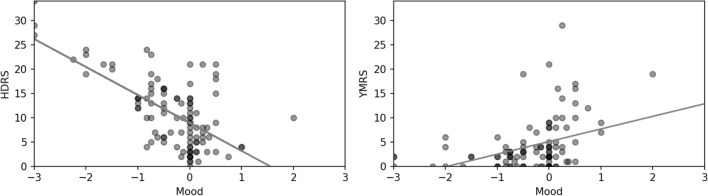
The association between Hamilton Depression Rating Scale 17-items score (HDRS), Young Mania Rating Scale (YMRS) and patient-reported smartphone-based data on mood. The grey line indicates the linear least square fit for each combination

Figure 2 presents the association between patient-reported mood and clinical ratings of depressive and manic symptoms according to the HDRS (r  = − 0.64, p  < 0.001) and the YMRS (r  = 0.39, p  < 0.001). In both cases the correlation coefficients were statistically significant.

**Fig. 3:**
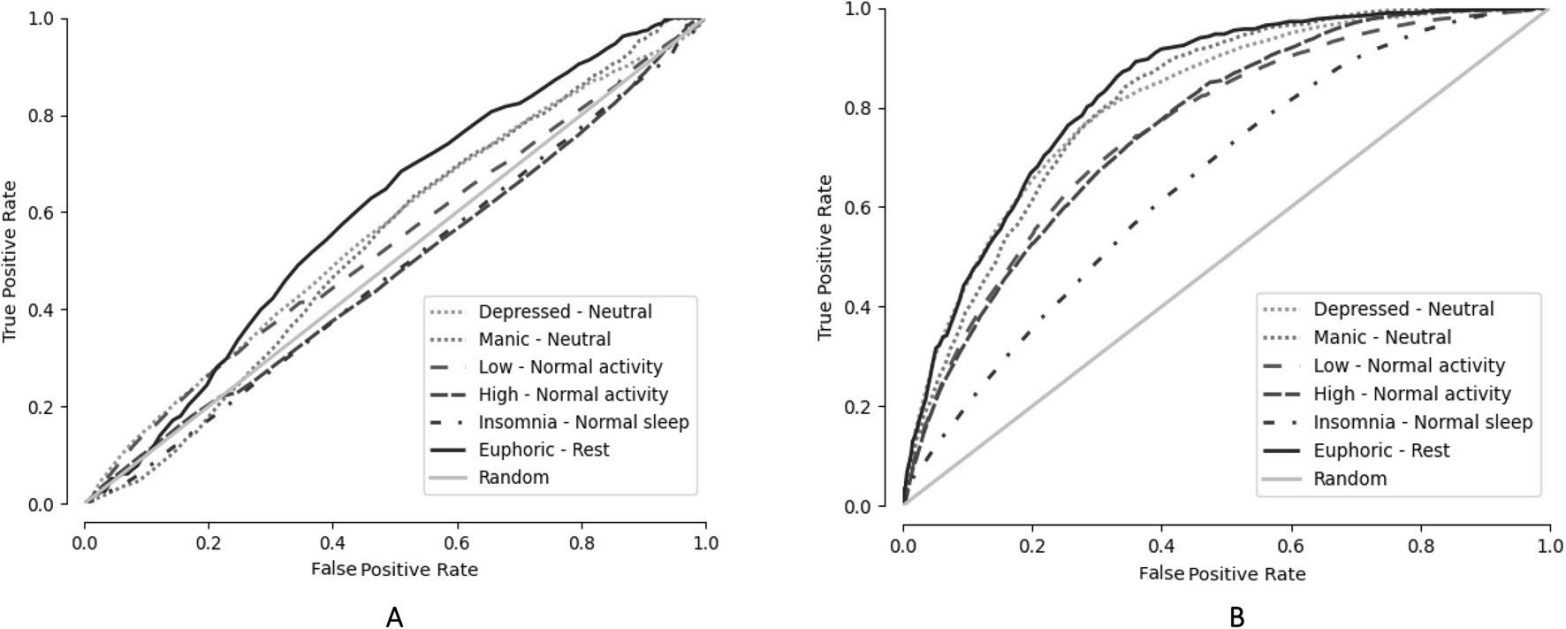
The ROC curve for the classifications of different states based on voice features in patients with bipolar disorder. **A** The user-independent models; **B** the user-dependent models. Euphoric defined as combined increased mood and increased activity

The ROC curves generated by aggregating all model estimates and the corresponding true class labels in each cross-validation fold, as well as each patient in the user-dependent classifiers, are presented in Fig. 3. As can be seen, the ROC curve for the sleep model is the closest to random, while the combined increased mood, increased activity and insomnia versus periods without performed best.

## Discussion

The present study investigated the use of voice features collected during naturalistic phone calls for classifications of patients with BD, HC and UR, as well as state classifications within patients with BD. First, and in accordance with our hypotheses, voice features rather sensitively discriminated BD from HC, but in contrast to our hypotheses with low specificity. Further, voice features statistically significantly differed between UR and HC, but in contrast to our hypotheses discriminated between the two groups with a modest accuracy.

Second, within patients with BD, voice features during mania (and to a lesser degree during depression) rather specifically discriminated from voice features during euthymia, but with low sensitivity. In addition, voice features during periods with insomnia quite specifically discriminated from periods without insomnia. The model including voice features during periods with combined increased mood, increased activity and insomnia performed best among all the models as reflected by the specificity and the AUC. In line with our previous study (Faurholt-Jepsen 2016), within patients with BD the user dependent models clearly performed the best for classifications of different states, suggesting that changes in voice features is individual, like ‘a fingerprint’.

Increased energy or activity has been highlighted in the DSM-5 and must now be present alongside mood changes to diagnose hypomania/mania raising energy/activity to criterion A (Kessing et al. 2021; Fredskild et al. 2021). Nevertheless, in contrast to our hypotheses, the sensitivity and specificity for discriminating between increased activity and neutral activity, and decreased activity and neutral activity was low.

Interestingly, the findings from the present study are in line with findings from previous studies suggesting that voice features may be used as a trait (Zhang et al. 2018) and state (Karam et al. 2014; Gideon et al. 2016; Vanello et al. 2012; Guidi et al. 2015; Faurholt-Jepsen 2016) marker in BD. However, previous studies included rather small samples of patients with BD and did not compare voice features to UR and HC.

A recent systematic review concerning automated assessment of psychiatric disorders using speech suggested that speech processing technology could aid mental health assessments (Low et al. 2020). Many people own and use a smartphone and smartphones comprise a unique platform for unobtrusive and continuous monitoring. Due to the limited access to treatment facilities, during the past 10 years, and especially during the COVID-19 pandemic, there has been an increase in the international interest in the use of mHealth technologies within mental health (Insel 2017; Wang et al. 2018; Anthes 2016; Patoz et al. 2021; Lagan 2020).

Within BD there is a substantial diagnostic delay, a progression of illness severity during untreated years, and a potential delayed intervention on subsyndromal symptoms. Assessments within BD are intermittent and may be limited partly due to the episodic nature of the disorder. The findings from the present study suggest that voice features may be used as an objective supplementary assessment method for diagnosis and identification of deterioration following initial informed consent. Such data has the additional advantage that they may be available when patients suffer from severe mood episodes and even when patients are non-adherent to treatment and don’t attend clinical appointments.

### Advantages and limitations

The present study was the first to include a large sample of both patients with BD, HC and UR, and is therefore hypothesis generating within the field. Furthermore, the patients with BD were followed for a long time period allowing for collection of both fine-grained voice and daily patient-reported data. The affective states within patients with BD were defined according to daily patient-reported smartphone-based data on mood, activity and sleep. In this way voice features and information on states was available for a larger proportion of days than if states were solely defined according to clinical ratings, which were conducted less frequently. While patient-reported smartphone-based mood was associated with scores on the HDRS and YMRS, a larger error margin was observed in the euthymic state (− 0.5 to 0.5). The ability for the model to discriminate from a euthymic state might be affected by the patient’s ability to self-assess when situated in the border between different states. Exploratory analyses investigating the classification of manic episodes using a cut-off on patient-reported smartphone-based mood  > 1 did not alter the estimates. Further, there may be a risk that the patient-reported smartphone-based data on mood were not missing at random, and thus voice feature during the most severe affective states might not have been included. The three included populations were well-characterized according to clinical as well as research-based assessments using the SCAN interview, and the patients with BD were newly diagnosed increasing the impact of the findings. A potential confounding effect of factors related to the mental health status of the included participants such as psychopharmacological treatment cannot be ruled out (Bock 2019). Future studies could consider investigating this aspect further.

In the present study, the sensitivity of discriminating between different affective states within BD was quite low, as reflected by the relatively modest AUCs. Considerations between the trade-off between the sensitivity and the specificity should be a priority in future studies.

The available voice features were collected unobtrusively during naturalistic settings reducing the Hawthorne effect (Wickström and Bendix 2000). The study included a large amount of fine-grained repeated data for each participant during long-term follow-up. However, in some of the analyses a low number of observations were included. The finding that within patients with BD, the user dependent models outperformed the user independent models, and the user independent models performed close to random, suggest that change in voice features are highly individual, and thus hard to generalize between individuals.

The present study included the Speech and Music Interpretation by Large-space Extraction (openSMILE emolarge) feature set. It is possible that other configurations of the openSMILE toolkit or other feature extraction technologies, and subsequent features selections, to the one used in the present study could be feasible while keeping or improving the classification. This would help to reduce computational costs and save storage space. Further, from the present study, it was not possible to extract which of the included voice features that were most contributing to the classification models. The present study included patients with BD, but not patients with other psychiatric disorders. Future studies investigating the use of voice features for differentiating between psychiatric disorders could provide exciting information within the area. The voice features were extracted during regular phone calls, and thus we did not have access to voice feature from communication using other smartphone-based platforms.

### Perspectives and future implications

Using voice features reflects a potential innovative, objective and unobtrusive supplementary method for discriminating patients with BD and UR from HC and as a state marker within patients with BD.

## Conclusions

The present study investigated for the first time the use of voice features collected during naturalistic phone calls in a large sample of patients with BD, HC and UR and for state classifications within BD. It was shown that voice features can discriminate BD from HC with high sensitivity, but with low specificity, and that voice features significantly can differentiate between UR and HC. Within patients with BD, mania was rather specifically discriminated from euthymia. However, the trade-off between the sensitivity and the specificity was in all models reflected by the modest AUCs.

Within patients with BD the user dependent models clearly performed the best for classifications of different states, suggesting that changes in voice features is individual, like ‘a fingerprint’. These results show that voice features collected during naturalistic phone calls could potentially be used as a supplementary objective marker discriminating patients with BD from HC and as a state marker within patients with BD.

## Data Availability

Not applicable.

## Abbreviations

BD: Bipolar disorder
UR: Unaffected first-degree relatives
HC: Healthy control individuals
SCAN: The Schedules of Clinical Assessment in Neuropsychiatry interview
HDRS: Hamilton Depression Rating Scale 17-items
YMRS: Young Mania Rating Scale
RF: Random Forest classifier
AUC: Area under the characteristic curve
ROC: Receiver operating characteristic
B10: Bayesian inference framework with intrinsic priors
M: Mean
SD: Standard deviation
